# Muscular Tuberculosis: A New Case and a Review of the Literature

**DOI:** 10.3389/fneur.2019.01031

**Published:** 2019-10-01

**Authors:** Yanping Zeng, Yin Liu, Yanchun Xie, Jingjing Liang, Jiabing Kuang, Zuneng Lu, Yu Zhou

**Affiliations:** ^1^Department of Neurology, Renmin Hospital, Wuhan University, Wuhan, China; ^2^Department of Orthopedic, Wuhan Fourth Hospital, Wuhan, China

**Keywords:** tuberculosis, muscle, muscular tuberculosis, biopsy, pathology

## Abstract

**Objective:** To investigate the clinical manifestations, underlying diseases, pathological features, and therapeutic responses in patients with muscular tuberculosis (MT), which is rare and often misdiagnosed in clinical practice.

**Methods:** This study describes a rare MT case that was recently diagnosed in our department. Additionally, 18 other MT cases retrieved from the PubMed database from 2,000 to date are included in this study. The clinical manifestations, areas of residence, underlying diseases, laboratory test results, pathology results, and outcomes of the patients were recorded and analyzed.

**Results:** The MT patients in this study included 13 males and six females with an average age of 34.58 years old. Eight patients were from Asia, and six patients were from Africa, accounting for the majority of patients, at 73.68%. Underlying diseases included flu-like illness in one patient; chronic kidney disease (CKD) in one patient; Sjögren's syndrome in one patient; systemic lupus erythematosus (SLE), lupus nephropathy, and deep vein thrombosis in one patient; and Alzheimer's disease (AD) and Paget's disease in one patient. Two patients had a previous history of tuberculosis, and two patients had contact with suspected tuberculosis patients. All patients presented chronic occult onset, with an average of 3 (1.75, 5) months. Six cases had local masses, and 13 cases had swelling as the main clinical manifestations. Twelve patients (63.2%) presented manifestations at single sites, and seven patients presented manifestations at multiple sites, including the thigh, calf, arm, chest wall, dorsal, psoas, gluteal, and forehead muscles. Of the 19 total patients, 13 (68.4%) reported pain, and only 8 (42.1%) patients presented tuberculosis symptoms. All patients received laboratory results associated with *Mycobacterium tuberculosis* infection. Fourteen (73.7%) of the 19 patients underwent skeletal muscle biopsy, where granulomatous inflammation was observed. Eighteen patients were treated with anti-tuberculosis therapies. Sixteen patients improved or recovered after anti-tuberculosis treatment, and unfortunately, two patients died.

**Conclusion:** As a kind of systemic disease, MT is mainly characterized by painful or painless muscle masses and swelling at a single site or at multiple sites. Patients with a history of tuberculosis and immune system disease are susceptible to MT. A diagnosis is mainly made on the basis of the results of pathological biopsy and bacteriological culture. Early diagnosis and timely standardized anti-tuberculosis treatment can improve the prognosis.

## Introduction

There are fewer and fewer tuberculosis patients globally because of the improvement of the economic level and the rational application of anti-tuberculosis drugs. In the past 20 years, the incidence of tuberculosis in most parts of the world has been declining, except for a significant increase in tuberculosis in sub-Saharan Africa. However, the control of tuberculosis has been threatened since the emergence of drug-resistant tuberculosis, and tuberculosis incidence has thus increased in some areas. The 2014 World Health Organization (WHO) Global Tuberculosis Report shows that almost half a million or more cases of tuberculosis occurred worldwide in 2013. Of the estimated 9 million people who developed tuberculosis in 2013, 1.5 million people died (deaths up from 1.3 million estimated in 2012) (1–3). The most common infection site of *Mycobacterium tuberculosis* is the lungs, followed by the lymph nodes, serosal cavity, digestive system, genitourinary system, etc., and oral tuberculosis, skin tuberculosis, bone tuberculosis, and nerve tuberculosis are relatively rare. Specifically, muscular tuberculosis (MT) is extremely rare and difficult to diagnose and distinguish in the clinic. To improve the understanding and diagnosis of MT, this study retrospectively analyzed 19 cases of MT, including general patient data, areas of residence, underlying diseases, pulmonary lesions, clinical manifestations, involved sites, various examination results, imaging results, pathology results, treatments, and outcomes, which are reported as follows.

## Patients And Methods

### Patients

We describe a patient with multiple MT involvement lower limbs who was hospitalized in the Neurology Department of Renming Hospital of Wuhan University in December 2018, and identified an additional 18 cases (17 articles) (4–20) in the PubMed database from 2000 to date using various search terms related to “muscular tuberculosis,” “muscle tuberculosis,” “musculoskeletal tuberculosis,” “skeletal muscle tuberculosis,” “tubercular myositis,” and “tubercular polymyositis.”

### Case Presentation

**Figure 1:**
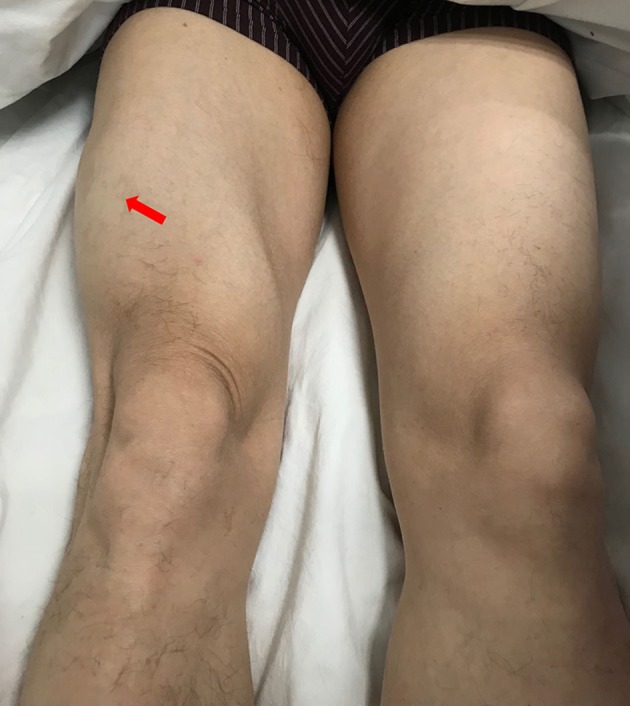
Clinical photograph showing a mass of left thigh without rash.

**Figure 2:**
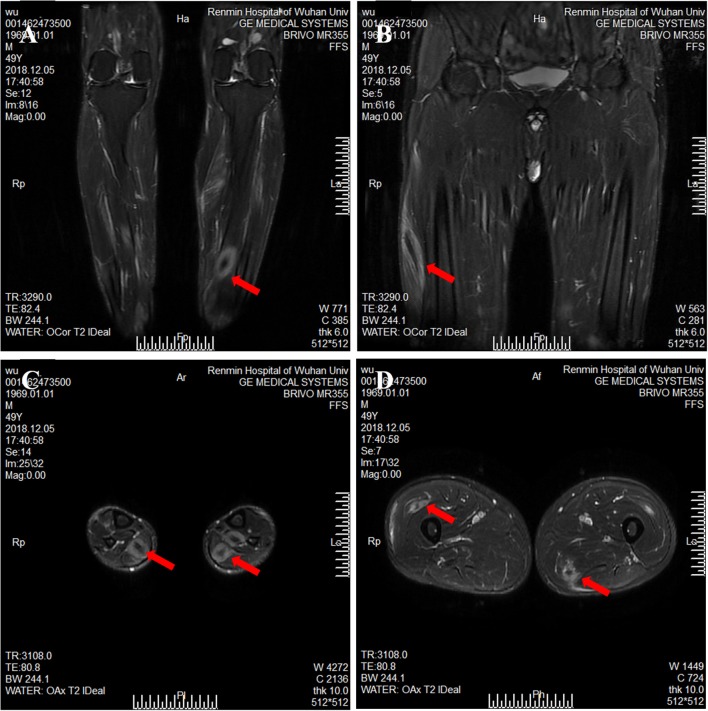
Magnetic resonance imaging of calf and thigh masses. **(A)** A coronal T2-weighted image shows a mass with a clear boundary, with a long T2 signal and a short T2 signal at the center of the mass in the calf. **(B)** A coronal T2-weighted image shows muscular enlargement with increasing signal intensity over the muscles and subcutaneous areas in the thigh. **(C)** The axial T2-weighted image showed a clear boundary mass with a long T2 signal and a short or equal T2 signal at the center of mass in the gastrocnemius and soleus muscle. **(D)** The axial T2-weighted image shows a mass with a blurry boundary, with a long T2 signal and a short T2 signal at the center of mass in the lateral femoral muscle and adductor muscle.

**Figure 3:**
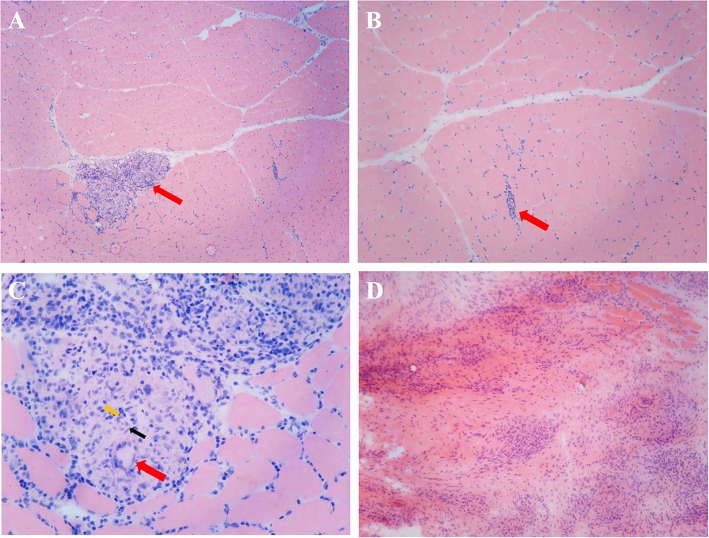
Histopathological examination of a mass and the nearby muscles. **(A)** Skeletal muscle HE staining, arrows indicate several well-formed inflammatory granulomas embedded among the muscle fibers (×50). **(B)** Skeletal muscle HE staining, arrows indicate inflammatory cell infiltration among the muscle fibers (×100). **(C)** Inflammatory granulomas embedded among the muscle fibers, red arrows indicate Langerhans cell, black arrows indicate epithelioid cells, and yellow arrows indicate lymphocytes (×400). **(D)** Inflammatory granulomas of the intramuscular mass (HE staining, ×100).

**Figure 4:**
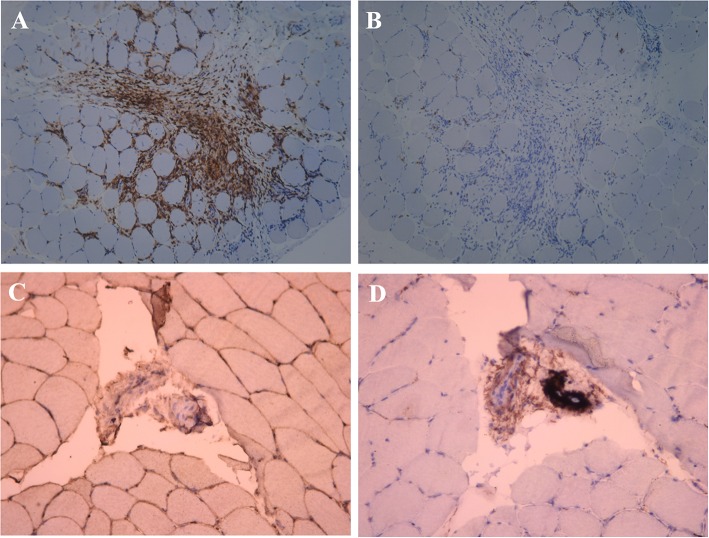
Immunohistochemical examination of the muscle near the mass. **(A)** Anti-CD4^+^ T cell monoclonal antibody immunohistochemical staining, positive expression of CD4^+^ in a large number of inflammatory cells (×100). **(B)** Anti-CD8^+^ T cell monoclonal antibody immunohistochemical staining, negative expression of CD8^+^ in the inflammatory cell (×100). **(C)** Anti-MHC-I monoclonal antibody immunohistochemical staining, MHC-I present on the sarcolemma (×200). **(D)** C5b-9 observed on the vessels (×200).

A 49-year-old male presented with a 9-month history of multiple anatomical site pain, a localized mass, and swelling of thighs (Figure 1) and calves, which gradually increased in size and quantity without systemic symptoms, such as fever, poor appetite, malaise, weight loss, or perspiration during sleep or after strenuous exercise. One month before admission, he complained of similar symptoms that occurred in the left forearm but to a lesser degree and with no mass present. He had a history of pulmonary tuberculosis. One year previously, the patient presented non-infectious posterior uveitis and had been treated with steroid for half a year. There were no systemic symptoms and no history of trauma, family history, or other disease history. His systemic physical examination was normal. Multiple anatomical sites on the thighs and calves contained masses, the borders of which were well-demarcated and cystic in consistency, but they were not fluctuant and there was no tenderness of the mass or increased local temperature. The skin over the mass was normal, with no rash observed. Musculoskeletal ultrasound examination suggested substantive bilateral lesions in the calves. Chest computed tomography (CT) revealed previous pulmonary tuberculosis. The patient underwent a musculoskeletal magnetic resonance imaging (MRI) examination in a local hospital; the results were reported as suggestive for rhabdomyolysis, only according to his history of strenuous exercise. The patient was admitted to the Department of Neurology, Wuhan University, Renmin Hospital, for diagnosis and treatment. Further examinations, including those for hemogram, rheumatoid factor (RF), C-reactive protein (CRP), erythrocyte sedimentation rate (ESR), creatine kinase (CK), aspartate aminotransferase (AST), alanine aminotransferase (ALT), aldolase, lactate dehydrogenase (LDH), fungal G-test, and tumor marker levels, were within normal limits. Anti-nuclear antibodies (ANA) were positive at a dilution of 1:100, and blood parasites were negative. Musculoskeletal MRI (Figure 2) examination showed diffuse abnormal signals on the bilateral calf and thigh muscles, suggesting infectious lesions or myopathy. Electromyography (EMG) examination showed that sensory and motor conduction are normal. There was no abnormal spontaneous activity in the anterior muscles of the double tibia and right medial thigh muscles. Paraspinal muscles [L5, S1] have little abnormal spontaneous activity. Motor unit potentials increased in duration and amplitude in the anterior muscles of the double tibia and right medial thigh muscles. It suggested nerve root damage of both lower limbs (may be root damage). In combination with the clinic, there is a great possibility of considering degeneration. A pelvis and lumbar spine MRI showed mild lumbar disc herniation without other abnormal findings. A deep biopsy (including skin, subcutis, fascia, and muscle) was performed on the right gastrocnemius (including the mass and nearby muscles), and the results showed lymphocytes and plasma cell infiltration into and around non-necrotic muscle fibers without muscle fiber necrosis and regeneration. Moreover, inflammation granulomas (including Langerhans cells, epithelioid cells, and lymphocytes) were visible under the microscope (Figure 3), and CD4(+), CD8(–), MHC-I(+), C5b-9(+) (Figure 4), and acid-fast stain(–) results were observed. Sputum examination revealed no evidence of tuberculosis bacilli, and a tuberculin skin test was negative. However, PCR for the *M. tuberculosis* complex presented positivity, and a tuberculosis T-SPOT test(+); *M. tuberculosis* was found in biopsy tissue culture of the mass on the calf. A CT scan revealed no bony erosion. A high-probability diagnosis of granulomatous myositis with *M. tuberculosis* infection in the thighs and calves was considered on the basis of clinical, MRI, culture, histopathology, and gene test results. Therefore, the patient was prescribed standard oral anti-tubercular treatment with four drugs, isoniazid, rifampicin, ethambutol, and pyrazinamide, for 2 months. After treatment, the patient's symptoms were relieved, and the swelling in the thighs and calves appeared to decrease in size. Further isoniazid and rifampicin were given. Currently, the patient is undergoing follow-up.

### Methods

We included articles describing patients with sufficient clinical and imaging detail that were published only in English and excluded an article with incomplete information. All cases were diagnosed with typical MT. Information of the cases was recorded and analyzed.

## Results

### Demographics and Clinical Characteristics

We found that 18 patients met our inclusion criteria. With the addition of our patient, a total of 19 cases were included in our descriptive analysis. Demographics and clinical characteristics are shown in Table 1, male/female = 2.17:1 with an average age of 34.58 ± 20.81 years old. Patients in Asian and African countries accounted for the majority (73.68%) of patients. There was underlying disease or a history of pulmonary tuberculosis in eight patients. All patients presented chronic occult onset, ranging from 2 weeks to 2 years, with an average of 3 (1.75, 5) months, and symptoms gradually aggravated. The patients mainly presented local masses, swelling, weakness, pain, or painlessness; six patients had a local mass as the main clinical manifestation, and 13 patients had swelling as the main clinical manifestation. Thirteen (68.4%) reported pain, while only 2 (10.53%) reported weakness. The skin was warm to the touch in one patient. Only eight (42.1%) patients had tuberculosis symptoms, including night sweats, weight loss, anorexia, fatigue, and intermittent fever. There were two patients with osteomyelitis. Twelve (63.2%) patients presented symptoms at single sites, and seven patients presented symptoms at multiple sites, including thighs, calves, arms, chest wall muscles, and so on (Table 1). One of the patients showed a left psoas abscess with involvement of the left iliac wing of the iliac crest consistent with osteomyelitis. In one patient, a CT scan showed soft tissue inflammation of the gastrocnemius, consistent with osteomyelitis of the proximal tibia. In one patient, the inter-pectoral space revealed a cystic abscess with necrotic material extending into breast tissue. In addition, one patient presented bony erosion.

**Table 1:** Demographics and clinical characteristics of 19 patients.

**Case**	**Gender**	**Age**	**Areas of residence**	**Underlying disease and pulmonary lesions**	**Main clinical manifestations**	**Involved sites**	**Other symptoms and signs**	**Course of disease**
1	M	49	Asia	Pulmonary tuberculosis in history, but normal now	Mass, swelling, pain	Both thighs, calves	–	9 months
2 (4)	M	23	Africa	A flu-like illness	Mass, pain, weakness	Forearm, thigh, calf	Tuberculosis symptoms	A few weeks
3 (5)	M	24	America	–	An enlarging soft-tissue mass, pain	Left psoas	Tuberculosis symptoms, osteomyelitis	5 weeks
4 (6)	M	49	Asia	CKD and steroid treatment	Mass, left leg pain, fever	Gluteus	–	1.5 months
5 (7)	F	9	Asia	–	Pain, swellings which were tender, fluctuant	Pectorales, forehead	Tuberculosis symptoms	8 weeks
6 (8)	M	53	Europe	Pulmonary tuberculosis in history, but normal now	A fixed tender mass	Pectorales	–	Chronic occult
7 (9)	M	55	Asia	Sjögren's Syndrome and steroid treatment	Swelling, tenderness, and localized heat	Right forearm, double thigh	Tuberculosis symptoms	3 months
8 (10)	M	71	Europe	–	Ill-defined mass, symptomatic, tender	Right thigh	Tuberculosis symptoms	6 months
9 (11)	M	15	Asia	–	Swelling, tender	Left thigh	Tuberculosis symptoms	UA
10 (12)	M	25	Africa	Exposed to suspected tuberculosis, diarrhea, weight loss	Enlarging swelling	Right gluteus	–	3 months
11 (13)	F	11	Asia	–	Swelling	Right thigh, right calf and left arm	–	4 months
12 (14)	M	25	Asia	–	Hard, fixed swelling	Right tricep	Tuberculosis symptoms	1 months
13 (15)	F	9	Asia	–	Swelling	Forearm, right calf	–	3 months
14 (16)	F	38	Africa	–	Pain and progressively increasing swelling	Dorsal muscles	Tuberculosis symptoms	2 months
15 (17)	M	24	Africa	–	Swelling and pain	Pectorales	–	2 years
16 (17)	M	38	Africa	–	Swelling and pain	Pectorales	–	2 years
17 (18)	F	33	America	SLE, Lupus nephropathy, hormonal therapy, left leg deep vein thrombosis	Pain, weakness, slight swelling, and redness	Right calf	Osteomyelitis	2 weeks
18 (19)	M	83	America	AD, Paget's disease, his mother died of tuberculosis when he was 2 years old	Swelling and tenderness	Anterior tibia muscle of left calf	–	UA
19 (20)	F	23	Africa		Swelling, pain	Left thigh	–	UA

–*, none; UA, unacquirable*.

### Laboratory Tests

The leukocyte counts and CRP and ESR levels were elevated in the majority of patients. Serum creatinase levels were normal or slightly elevated. All patients received laboratory results associated with *M. tuberculosis* infection; laboratory analyses included one or more of the following: a tuberculin skin test, an acid-fast bacilli smear, a tissue culture, a tuberculosis infection T-SPOT test, PCR for the *M. tuberculosis* complex, and a gene Xpert MTB/RIF assay of the biopsy.

### Imaging Findings

The patient's imaging examinations included ultrasound imaging, CT evaluation, and/or MRI. Ultrasound results showed cystic lesions with thick irregular walls and single or multiple nodules in the intramuscular layer. CT results showed loculated low-density collections. MRI showed well-defined masses in involved muscles that were hyperintense on T2-weighted images and hypointense on T1-weighted images, with peripheral ring hyperintensity after the injection of a gadolinium contrast agent.

### Pathology

Fourteen (73.7%) of the 19 patients underwent skeletal muscle biopsy. After the specimens were fast frozen as slices or paraffin sections and haematoxylin and eosin (HE) stained, the main observation under the microscope was inflammatory infiltration, mostly by lymphocytes. In some regions, granulomatous inflammation, including epithelioid histiocytes, Langerhans giant cells, and caseating necrosis, was observed singly or in combination.

### Differential Diagnosis

In addition to tuberculosis, granulomatosis includes fungal and parasitic infections, sarcoidosis, lymphomatoid granuloma, and so on. However, the blood fungus G test and Gomori methenamine silver (GMS) stain were negative, and the blood parasite was negative in the patient. Sarcoidosis and tuberculosis are similar histologically, but they are slightly different. There are a lot of epithelioid cells in nodules of sarcoidosis, and few lymphocytes, but obvious lymphocyte infiltration around epithelioid cells in nodules of tuberculosis. There are blood vessels in nodules of sarcoidosis, and necrosis seldom occurs; but there are no blood vessels in nodules of tuberculosis, so caseous necrosis often occurs. Lymphomatoid granuloma is a vascular-central invasive disease, also known as vascular-central lymphoma.

### Treatments and Outcomes

Except for one case, the treatment and prognosis of patients were not recorded in detail. Eighteen patients were treated with anti-tuberculosis therapy. Seventeen patients were mainly treated with the following first-line anti-tuberculosis chemotherapy drugs: isoniazid (INH), rifampicin (RIF), ethambutol (EMB), and pyrazinamide (PZA), with three or four combined therapies for 2 months, followed by INH and RIF therapy. Sixteen patients improved or recovered after anti-tuberculosis treatment, and unfortunately, two patients died. On the fourth day of anti-tuberculosis chemotherapy, one patient's fever began to resolve, and the pain started to gradually diminish; however, unfortunately, the patient died of cerebrovascular hemorrhage during the third week of anti-tuberculosis chemotherapy. The other patient died of profound shock and multiorgan failure on his 18th day of hospitalization.

## Discussion

Recently, the incidence of tuberculosis has rebounded, and extra-pulmonary tuberculosis has become common. MT is rare and has been reported in many countries. All MT cases included in this analysis were case reports, except for those by Gou et al. (21) and Wang et al. (22). We reported a case of a MT patient who we recently treated, and we analyzed the English literature from 2000 to date; the results are discussed as follows.

According to the reports of domestic and foreign literature, there is no gender difference in the incidence rate of MT. Gou et al. (21) reported a male:female gender ratio of 16 (46%):19, and a previous male:female gender ratio of 8 (42%):11; however, the male:female ratio = 13 (68.42%):6 in our analysis of the literature, with no significant differences (*P* = 0.11). All ages presented morbidity, and the average age of the 19 patients who we analyzed was 35 years (9–83 years), which was consistent with the average age of 38 years (0.7–86 years) reported by Wang et al. (22).

Tuberculosis is common in undeveloped or developing countries, but no literature has reported regional differences in the rates of extra-pulmonary tuberculosis, especially MT. The English literature cases we studied were from all over the world, and there were certain geographical differences. MT patients accounted for the vast majority, with rates as high as 73.68% in Asian and African countries.

The patient we reported here had a history of tuberculosis many years ago and uveitis in the eye 1 year previously. Because of the uveitis, the patient was treated with steroid therapy; therefore, the body's immunity was reduced. It is possible that latent tuberculosis of the lungs spread to the muscles, resulting in MT. Adrenal corticosteroids have inhibitory effects on many aspects of the immune process. The patient had impaired immune function due to the administration of steroidal drugs, providing an opportunity for the invasion and reproduction of *M. tuberculosis*. The patient's purified protein derivative (PPD) skin test was negative, which was also a manifestation of the inhibition of immunity. Furthermore, we reviewed the pathogenesis of tuberculosis infection and found that the majority of tuberculous primary lesions had formed in patients when they were juveniles, and the tuberculosis had reached the lungs by the first respiratory infection. During this time, the body has not yet developed specific immunity, and the pathogen travels along the lymphatic vessels to the hilar lymph nodes and then enters the blood, resulting in primary bacteremia. After 4–6 weeks, body-specific immunity is formed, and more than 90% of the primary tuberculosis is eliminated. However, some patients develop minute lesions that are in a static state. When the immune function of the body is reduced, the resting lesions are activated, forming lesions presenting local symptoms, signs, and systemic reactions. At present, it is generally agreed that MT spreads hematogenously; however, it has been reported that patients with MT and intestinal tuberculosis show abundant lymphoid tissue at the ileum end and large intestine. Therefore, whether MT is related to lymphoid tissue that engulfs and transports *M. tuberculosis* and causes local deposition of *M. tuberculosis* in the muscle through lymphatic circulation is worth exploring. We found that those patients who have been treated with steroid have decreased immunity and that those who have a tuberculosis infection or exposure history are susceptible to MT, which is consistent with the results of Gou et al. (21) and Wang et al. (22). The route of infection can be exogenous or endogenous. The former is associated with a history of trauma, and the latter is caused by infection of the blood and lymph and direct infiltration of muscle.

MT presented a chronic occult onset and was gradually aggravated. The main clinical manifestations of the patients were local masses, swelling, weakness, and pain or painlessness. In this study, less than half of patients (42%) had systemic tuberculosis symptoms, and the systemic symptoms reported by Wang et al. (22) were even less, occurring in only eight (22.9%) patients. The symptoms and signs of MT patients are not specific and suggestive, resulting in certain diagnostic difficulties.

MT is rare because the muscle tissue has increased blood circulation, and the lactic acid produced during exercise kills *M. tuberculosis*. However, when the body's immune function declines, latent *M. tuberculosis* in the body spreads through the blood to form tuberculosis lesions throughout the body. In addition, due to other reasons, including trauma, lymphatic dissemination of *M. tuberculosis*, and direct infiltration, systemic muscle tissue in any part of the body can be affected. In our 19 patients, the limbs were involved more often than others body parts, and the lower limbs were more common, which was consistent with the literature (21, 22). However, Gou et al. (21) reported the most involvement in calf tissues, and in all of our cases, the thigh was the most affected; therefore, more cases should be analyzed in the future. According to all current case reports, muscle involvement in the extremities is most common, followed by involvement of the trunk muscles and muscles in the head and neck. Most of the patients in this study had muscle involvement at a single site, which was consistent with previous reports.

The diagnosis of MT mainly depends on pathological biopsy and bacteriological culture. Of the 19 patients reported in this study, 14 were diagnosed by pathological findings of granulomas. Gou et al. (21) reported that 14 patients in their study were diagnosed by pathological findings of granulomas. All patients presented evidence of tuberculosis infection, detected by one or more of the following: a tuberculin skin test, an acid-fast bacilli smear, tissue culture, a tuberculosis infection T-SPOT test, PCR for the *M. tuberculosis* complex, and a gene Xpert MTB/RIF assay of the biopsy. The tuberculosis test can be partially negative, which does not rule out tuberculosis infection. Similarly, a lack of granuloma does not exclude the diagnosis, especially in highly suspicious cases. In the laboratory, inflammation indicators are not necessarily elevated, and ESR in some patients is within normal limits. Imaging studies, including ultrasound, CT, and MRI, can identify lesions and locate biopsy locations.

The main treatments are standardized anti-tuberculosis therapies and lesion removal. For tuberculous nodules in single sites, surgical resection can be performed, but multiple nodules in multiple sites cannot be completely surgically removed. Overall, the standardized application of tuberculosis drugs and anti-tuberculosis treatments has provided good prognoses for MT patients in recent decades. In our study, 16 patients improved or recovered after anti-tuberculosis treatment, and unfortunately, two patients died. The reason for poor prognosis is mainly due to other complications, such as cerebrovascular hemorrhage, shock, and multiorgan failure. We found that the prognosis of patients treated with hormones is worse than that of patients not treated with hormones. In the study by Wang et al. (22), the mortality rate was 14.3% and was even higher (30%) in patients with hematogenous tuberculous myositis. They reported the following reasons for the high mortality rate: the use of corticosteroids, a high *Mycobacterium* load, multiple muscle involvement, and large abscess formation. We consider that mortality may also be associated with early diagnosis and timely anti-tuberculosis treatment.

## Conclusion

MT is clinically rare and can be easily misdiagnosed because of its atypical and non-specific symptoms. However, MT should be considered in a differential diagnosis when patients present with swelling and pain of the soft tissue, and especially with single or multiple intramuscular masses. The gold standards for diagnosis are pathological biopsy and bacteriological culture. Early diagnosis and timely standardized anti-tuberculosis treatment can improve the prognosis.

## Data Availability Statement

All datasets generated for this study are included in the manuscript/supplementary files.

## Ethics Statement

This study was approved by the ethics committee of the Remin Hospital of Wuhan University, Wuhan, China. Written informed consent was obtained from the participant for the publication of the data and any accompanying images. All the figures referred to our case report.

## Author Contributions

ZL and YZh designed research. YZe and YL carried out it. YX analyzed results. JL and JK assisted with YZe wrote the manuscript.

### Conflict of Interest

The authors declare that the research was conducted in the absence of any commercial or financial relationships that could be construed as a potential conflict of interest.
